# Biomarkers for hazard identification in humans

**DOI:** 10.1186/1476-069X-10-S1-S11

**Published:** 2011-04-05

**Authors:** Helmut Bartsch, Khelifa Arab, Jagadeesan Nair

**Affiliations:** 1 Division of Toxicology and Cancer Risk Factors, German Cancer Research Center (DKFZ)Im Neuenheimer Feld 280, 69120 Heidelberg, Germany; 2 Division of Epigenomics and Cancer Risk Factors, German Cancer Research Center (DKFZ)Im Neuenheimer Feld 280, 69120 Heidelberg, Germany

## Abstract

**Background:**

Oxidative stress enhances lipid peroxidation (LPO), which both are implicated in the promotion and progression stages of carcinogenesis, in particular under conditions of chronic inflammation and infections. Exocyclic etheno-DNA adducts, which are formed by LPO-products such as 4-hydroxy –2-nonenal, are strongly pro-mutagenic DNA lesions.

**Methods:**

The development of ultra-sensitive detection methods for etheno-adducts in human tissues, white blood cells( WBC) and urine has provided evidence that these adducts are elevated in affected organs of cancer-prone patients, probably acting as a driving force to malignancy.

**Results:**

Two recent studies that yielded some new insights into disease causation are briefly reviewed:DNA-damage in WBC of mother-newborn child pairs, and lipid peroxidation derived DNA damage in patients with cancer-prone liver diseases. Our results indicate that biomonitoring of etheno-DNA adducts in humans are promising tools (*i*) to better understand disease aetiopathogenesis, allowing hazard identification(*ii*) to monitor disease progression and (*iii*) to verify the efficacy of chemopreventive and therapeutic interventions .Such clinical trials are warranted.

## Background

Oxidative stress enhances lipid peroxidation (LPO), which both are implicated in the promotion and progression of chronic degenerative diseases (CDD) , including carcinogenesis, in particular under conditions of chronic inflammation [1]. Common pathways of CDD involve biologically reactive oxygen and nitrogen species which can be generated by biochemical redox reactions and stress response enzymes such as lipoxygenases, cyclooxygenenase -2 and inducible nitric oxide synthase [2]. The resulting oxidative stress is currently implicated in over 100 human and animal CDD.Exocyclic etheno-DNA adducts, which are formed by LPO- products such as 4-hydroxy-2-nonenal (HNE), are strongly miscoding, causing specific point mutations. The development of ultra-sensitive detection methods for etheno-adducts in human tissues, WBC and urine provided evidence that these adducts are elevated in affected organs of cancer-prone patients, suggesting that such DNA damage can drive cells to malignancy [3].

**Figure 1:**
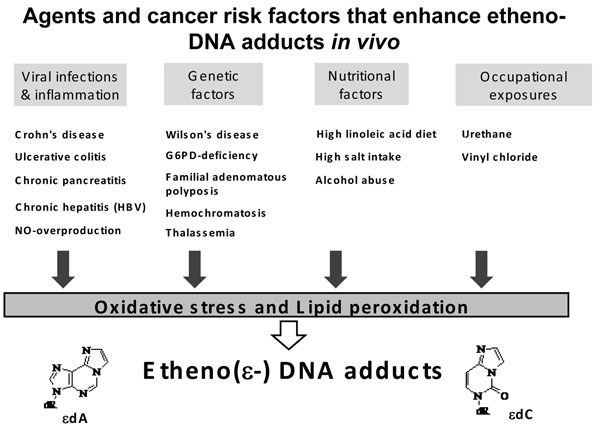
Studies that have demonstrated an increase in etheno-DNA adduct levels due to carcinogen exposure, acquired or inherited cancer risk factors. DNA – damage occurs as a consequence of oxidative stress and LPO as shown in humans and rodent models (reviewed in [1,2] ).

A summary of studies that we have conducted is depicted in Fig. 1; detailed adduct levels and results ( published until 2007 ) are reviewed in reference [1]. Sources of DNA damage that significantly increased the physiological background of exocyclic etheno-DNA adducts were found to be known human cancer risk factors such as i) viral infections and chronic inflammatory processes, ii) genetic cancer predisposing diseases, iii) nutritional factors and iv) certain occupational exposures. The recent study in a Danish cohort of mother- newborn child pairs is separately dealt with.

In the following, recent studies in humans that yielded some new insights into disease causation are briefly reviewed: 1) DNA -damage in WBC DNA of mother- newborn child pairs [4], and 2) Lipid peroxidation-derived DNA damage in patients with cancer-prone liver diseases [5]. Our results indicate that biomonitoring of etheno-DNA adducts in humans are promising tools (i) to better understand disease aetiopathogenesis, allowing hazard identification, especially when combined with genetic predisposition studies( e.g.as done in patients with metal storage diseases), (ii) to monitor diagnostic disease progression and (iii) to verify the efficacy of chemopreventive and therapeutic interventions. Such clinical trials are warranted and feasible due to the available non-invasive biomarkers we have developed.

## Methods

We have developed ultrasensitive, specific and artifact- free detection methods using immunoenrichment that allow the biomonitoring of etheno DNA adducts in humans *in vivo*[1]. Protocols and technical details are described in [6]. The high sensitivity and specificity of the three methods (below) are due to the use of monoclonal antibodies. They recognize etheno-DNA adducts specifically, allowing a) their immunopurification and quantitation in human tissues, cells and in urine where the adducts are excreted ; and b) their semiquantative immunohistochemical detection in tissue mircoarrays.

-	Adducts can now be quantified in tissue- and WBC-DNA by an ultrasensitive immunoaffinity-^32^P -postlabeling method.

-	Etheno-adducts excreted in urine can be measured by an immuno-enrichment-HPLC-fluorescence method.

-	Immunohistochemical staining in tissue sections allows to assess the prevalence of etheno-adducts in the nuclei of various cells and tissue types.

## Results

### Typical signature of exocyclic DNA damage measured in a cohort of mother-newborn child pairs

DNA damage is commonly thought to be involved in CDD causation, and its impact is particularly detrimental during fetal development. Hereby it is assumed that oxidative stress-related damage targets stem cells and their progenitors during differentiation, and that these early induced DNA lesions could cause hematopoietic disorders later in life. Within a multicenter study, we have analyzed 77 samples from mother-newborn child pairs to see if imprinting of DNA damage in mother child shows a similar pattern; it is known that cord blood is highly enriched with stem cells. Two etheno DNA-adducts, εdA and εdC, were measured and their levels (expressed per 10^9^ parent nucleotides) in leucocytes varied from 400-9000, demonstrating that even the lowest levels could be reliably quantified by our detection method. In child cord blood adduct levels were found to be about two times lower than in maternal blood ,the difference being highly significant. Analysis of εdA and εdC levels in cord versus maternal blood WBC showed strong positive correlations (R^2^ = 0.9, P <0.00001). Our results confirm that the DNA damage observed in leucocytes, arises from LPO-derived reactive aldehydes such as HNE, and suggests that a typical signature of DNA damage is induced similarly in fetus and mother [4]. Prospective cohort studies have to reveal whether these exocyclic WBC-DNA adducts could serve as predictive hazard indicator for developing hematopoietic cancers and other disorders later in life. Attention should be focused on outliers with extremely high adduct levels that were also found in this study. Analyses by the study consortium whether specific exposures measured in these populations are related to etheno-adduct formation are in progress.

### Lipid peroxidation-derived DNA adducts: potential early hazard markers in patients with liver cancer -prone diseases

Chronic alcohol abuse and hepatitis B, and C virus (HBV, HCV) infection are causally associated with increased risk of developing hepatocellular carcinoma (HCC) which progresses through chronic hepatitis, fibrosis and cirrhosis. The disease progression is associated with intense oxidative stress in the liver, resulting in an overproduction of reactive oxygen /nitrogen species and LPO-products [7].

Our aims of the study were to ascertain whether (*i*) etheno-adducts are formed in the liver of HCC-prone patients, (*ii*) etheno-adducts (εdA) areexcreted in urine and (*iii*) these adducts could serve as DNA damage (and putative risk) marker during viral -and alcohol -related liver cancer progression.

#### Studies in ALD patients

We analyzed εdA levels in DNA of needle liver biopsies obtained from European ALD patients diagnosed with alcohol-related hepatitis, fibrosis and cirrhosis using immunohistochemistry [8]. Cell nuclei positively stained for εdA were counted and the % prevalence was monitored. When compared to asymptomatic livers, the mean prevalence values were 16 times higher in biopsies from fibrosis/cirrhosis patients. This increase was comparable to that found in liver of patients with primary hemochromatosis, a genetically inherited disease highly predisposing to HCC. In European ALD patients, we also found in urine a 10 fold increased εdA -level as compared to healthy controls [9]. Thus high urinary εdA -excretion mirrors the massive DNA damage generated in the liver of ALD patients.

Excessive etheno-DNA adducts found in ALD patients are thought to be formed by the reaction of the major LPO product 4-hydroxy-2-nonenal (HNE) with nucleobases [5]. Confirmation for such a pathway in human liver *in vivo* was for the first time obtained by showing that protein- bound HNE and etheno adducts in ALD liver biopsies were strongly intercorrelated . In addition both markers correlated with CYP2E1 expression (R ^2^=0.9, P =0.01)

Our data support that ethanol-mediated induction of hepatic CYP2E1, leading to miscoding LPO-derived DNA lesions plays a central role in hepatocarcinogenesis, particularly in Caucasian ALD patients.

#### Pilot studies in virus-infected patients

As found in ALD patients, also subjects with inflammatory cancer -prone liver diseases caused by viral infection excreted high levels of εdA in urine [9]. Thai patients with chronic hepatitis, liver cirrhosis and HCC due to HBV-infection had 20, 73 and 39 times higher urinary εdA-levels, respectively when compared with asymptomatic HBsAg-carriers. In over two thirds of European patients with HBV and HCV -related liver disease, urinary εdA levels were increased 6 to 10 fold [9].

From these studies we conclude that (i) high urinary εdA-levels reflecting massive LPO-derived DNA-damage *in vivo* may contribute to the development HCC; (ii) εdA-measurments in urine and needle biopsies from target tissues in affected patients should be explored as a putative risk markers to follow malignant progression of inflammatory liver diseases and (iii) etheno adducts in urine may serve as non- invasive markers to assess to efficacy of chemopreventive and therapeutic interventions.

## Discussion

Since more than two decades, starting under the directorship of Lorenzo Tomatis (from1982-1993) at IARC in Lyon we pursued the following hypothesis: Major risk factors for various epithelial human cancers are endogenously produced DNA reactive species , that arise from dietary imbalance, chronic inflammation, persistent infections and aberrant metal storage. These damaging agents escaped detection by conventional biomonitoring and epidemiological techniques. Therefore new approaches in molecular epidemiology are required that include non- invasive methods for identification of such DNA damage in humans. We contributed to this paradigm by studying modified DNA base adducts that are generated by reactions of DNA with LPO-products, such as HNE that yield etheno-adducts for which we have developed ultrasensitive detection methods.

We then investigated pathological conditions and known cancer risk factors that could significantly increase the physiological background of etheno-adduct levels. A summary of our previous studies is depicted in Fig. 1 . Our recent mother- newborn child study is not ncluded as we are unable to associate specific exposures/ life style factors with etheno adduct formation in these WBC. In this biomonitoring study we detected highly variable, and possibly cancer-relevant DNA damage in WBC-DNA of mother-newborn child pairs, conceivably derived from dietary imbalance and life style factors. One source of etheno adduct may be explained by our previous findings that high dietary omega-6 polyunsaturated fatty acid (linoleic acid) intake drastically increased the formation of etheno-DNA adducts in white blood cells of female but not of male subjects [10]. In our mother –child study the DNA damage observed in maternal WBC-DNA was found to be very similar in DNA of newborns. In addition (epi)genome - environment interactions need to be further studied, so to allow better disease risk evaluations and predictions in children.

Our other investigations in cancer prone patients found progressively increasing DNA damage in preneoplastic liver tissue, due to alcohol abuse and viral infections, both causing chronic inflammation cascades that impose persistent oxidative stress in this organ.As published recently, tobacco smoking and UV- light induced many thousands of sequence alterations and specific signatures of somatic mutations in human lung cancer and melanoma [11,12]. Exocyclic LPO- derived DNA-lesions are likely to contribute to the mutation load reported to occur in human tumors.

Outlook: Since the discontinuation of our research project at DKFZ, there are a number of issues in relation to the utility of etheno adducts as biomarkers for hazard identification that need to be resolved:

Only three studiesin humans( Fig.1) have shown a positive relationship between etheno adduct formation and exposure to *specific agents*: Linoleic acid(omega-6 PUFA), sodium chloride and alcohol.

In contrast to rodent studies, only a few dose-reponse curves but mostly high *vs* low dose (intake) comparisons were done. Etheno adducts in urine of Japanese subjects living in a high risk area for gastric cancer were positively associated with the excretion sodium chloride ( a risk factor for gastric cancer) and omega-6 polyunsaturated fatty acid intake .

The time window was mostly investigated in rodent cancer models. In cancer prone-patients etheno adduct levels progressively increased in target organs , being highest in preneoplastic lesions with subsequent decline in the tumor tissue, the same time course as also seen in rodents.

The diagnostic and prognostic value of etheno adducts ,measured in needle tissue biopsies, white blood cells and as urinary excretion products needs to be established. Initial attempts to develop an inexpensive and simple kit for adduct analysis to allow easy application in clinical and epidemiologial studies, was not successful. Ultrasensitive, specific mass- spectrometric methods that have become available, should augur progress in this validation process , allowing high throughput work flow for large cohort analyses.

## Conclusions

From consistent results obtained in our past conducted studies ( Fig.1) we conclude:

(i)	Etheno- adducts,stable DNA damage markers are elevated by various cancer risk factors that impose oxidative/ nitrosative stress and LPO on a cell.

(ii)	Etheno-adducts progressively increased in many target organs and preneoplastic lesions of cancer prone-patients and rodents.

(iii)	LPO-derived DNA adducts appear to be one of the causes of mutations and genomic instability that drives carcinogenesis, offering promising markers for quantifying increasing loads of promutagenic DNA damage in early stages of carcinogensis.

(iv)	Etheno adducts in should be useful for etiology studies on cancers with ill-defined mechanisms, such as childhood malignancies: High DNA damage was found in some newborn child -mother pairs for which the cause is unknown.

From our findings in liver cancer prone patients we conclude:

(i)	High urinary εdA -levels reflecting massive LPO- derived DNA damage in the affected liver, may contribute to the initiation and progression of HCC.

(ii)	Measurements of etheno adducts in urine and in needle liver biopsies should be explored as a putative risk markers to follow the malignant disease progression in high risk patients patients and due to ALD and the viral infections.

(iii)	Etheno adducts as biomarkers hold promise to assess the efficacy of chemopreventive and therapeutic interventions and such clinical/biomonitoring trials are warranted.

## List of abbreviations

εdA: 1,*N*^6^ -ethenodeoxyadenosine; εdC: 3,*N*^4^-ethenodeoxycytine; ALD: alcohol-related disease; HBV: hepatitis B virus; HCV: hepatitis C virus; HCC: hepatocellar carcinoma; HNE: 4-hydroxy-2-nonenal; CYP2E1: Cytochrome 2E1; ROS: reactive oxygen species; LPO: lipid peroxidation; CDD: chronic degenerative diseases
